# Plasma-Activated Tap Water with Oxidative Potential Has an Inactivating Effect on Microbiological Contaminants in Aqueous Suspensions

**DOI:** 10.3390/pathogens13070535

**Published:** 2024-06-24

**Authors:** Nahla C. Droste, Mareike Hummert, Paul Leenders, Alexander Mellmann, Karsten Becker, Thorsten Kuczius

**Affiliations:** 1 Institute of Hygiene, University Hospital Münster, Robert Koch-Straße 41, 48149 Münster, Germany; nahla.droste@uni-muenster.de (N.C.D.); hummertm@uni-muenster.de (M.H.); alexander.mellmann@ukmuenster.de (A.M.); 2 VitalFluid BV, High Tech Campus 25-5, 5656 AE Eindhoven, The Netherlands; paul.leenders@vitalfluid.com; 3 Friedrich Loeffler-Institute of Medical Microbiology, University Medicine Greifswald, Ferdinand-Sauerbruch-Straße 1, 17475 Greifswald, Germany; karsten.becker@med.uni-greifswald.de

**Keywords:** plasma-activated water, antimicrobial effect, reactive species, watery environment, *E. coli*, *Enterobacterales*, waterborne microorganisms

## Abstract

Plasma-activated water (PAW) generated from tap water has gained attention as a disinfectant when used directly in its pure form. Little is known about the application of PAW for bacterial inactivation in aqueous environments because its use in fluids results in dilutions. We investigated the effect of PAW in aqueous suspensions simulating such dilutions, and we focused on the minimal addition of PAW volumes to bacterial aqueous suspensions still resulting in high inactivation rates. The antimicrobial effect was highly dependent on the activation of PAW. An increase in activation power from 90 to 100 W resulted in a greater microbial reduction with an identical 10 min activation time. The susceptibility to PAW dilutions was analyzed in detail regarding nine Gram-negative species out of *Enterobacterales* and other waterborne microorganisms as well as four Gram-positive species present in two different matrices, in saline and in tap water, at high concentrations simulating massive contamination situations. For this purpose, the PAW activation setting of 90 W and 30 min was defined in order to be able to differentiate the limitations of inactivation in individual bacterial species. The Gram-negatives in saline demonstrated susceptibility when one volume unit of PAW was added. However, twice the PAW volume was necessary for inactivation when bacteria were present in tap water. Gram-positive microorganisms were more robust, indicated by prolonged contact times before inactivation. Our results indicate that PAW can be used for bacterial decontamination processes in aqueous environments when added in surplus. Optimized activation settings such as electric power to generate PAW and the contact times to the samples increase the effect of the inactivation a wide range of bacteria, regardless of their resistance profiles.

## 1. Introduction

The demand for novel disinfection methods has steadily increased in recent years. Precautions to protect against microbiological and viral contaminations have become highly relevant, especially in the medical and food sectors, but also in the urban and rural environment, primarily regarding public health. Such disinfectants should address new challenges to protection against the emergence of new and increased pathogenic and antibiotic-resistant microorganisms. These have evolved through environmental conditions with high stabilities and tolerances to environmental stress situations and the rise of resistances to antibiotics [1]. Moreover, the emergence of biocide- and heavy metal/metalloid-resistant or -tolerant organisms may lead to mutual co-selection and additionally aggravates infection control and limits treatment options [2,3,4].

In the search for new disinfection processes, plasma technology has proved to be very suitable. Non-thermal sources use electrical discharges as a dielectric barrier, corona, spark, and underwater discharges, as well as atmospheric pressure plasma jets and plasma torch [5,6]. Novel alternative biocidal agents, which are highly active, can easily be produced by this technique. Various types of liquids have been plasma-activated as solutions (phosphate-buffered saline and saline) [7], oils [8,9] and different water types such as distilled [10,11], deionized [12,13], and tap water [14,15]. Plasma-activated water (PAW) proved to be biocidally effective against microorganisms [16,17,18], biofilms [19,20], and yeasts [21,22] in numerous applications. This led to the development of a variety of reactors with various operating principles to produce PAW [5,23,24]. In general, the basis for PAW generation is the interaction of water with plasma above or below the surface. PAW acts as an aqueous biocidal agent while oxygen and nitrogen in the air as well as in the water dissociate followed by further reactions of the dissociated and non-dissociated molecules to long-living and short-living reactive oxygen and nitrogen species (RONS) [18,25]. RONS in PAW initiate a pH decrease and an electrical conductivity increase [26,27]. Hydrogen peroxide and ozone are some of the main reactive oxygen species [28,29], and nitrate, nitrite, and peroxynitrite are among the reactive long-living nitrogen species [30,31]. The RONS formed during plasma activation appear to play a dominant role in antibacterial efficacy, as further chemical reactions between the reactive species and water follow, causing further reactions with the generation of different primary and secondary species. The oxidative reactive components originating from PAW generation cause oxidative stress on microorganisms with the consequence of membrane damage and consequently damage of intracellular components [26,32]. The damaged membrane integrity forms pores enabling the flow of intracellular components starting with small ions followed by larger molecules including nucleic acids and proteins [6], which leads to the oxidation of lipids and proteins [33,34]. 

PAW is an environmental and economical friendly disinfectant compared with chemical products, which may break down into by-products that are harmful to the environment and to public health [35]. Using devices on demand, PAW can be produced with several activation powers varying in voltage and activation time and in different volumes as required. This technology is applied in the decontamination of food products [36,37,38], where PAW is mainly used directly as decontamination liquid.

In contrast, little is known about the application of PAW for the decontamination of fluids such as tap water and the associated distribution pipes, which could be detrimental, as this is always accompanied by a PAW dilution effect. The aim of this study was to investigate the antimicrobial properties of PAW generated from local tap water in aqueous suspensions enriched with high bacterial loads. We focused on the application of PAW for the decontamination of contaminated fluids to which PAW was added in certain volume units resulting in a dilution effect of PAW. In order to identify bacterial susceptibilities and robustness of different microorganisms and species in solutions to PAW treatment, the minimal but necessary PAW generator’s settings were determined and the physical and chemical properties of the PAW and the contact times with the bacterial solutions were analyzed. These settings were applied to the respective environmental conditions. The inactivation efficacy was tested with a challenging collection of Gram-negative and Gram-positive microorganisms in the planktonic state present in tap water and in saline solution. Our results indicate the successful inactivation of microorganisms present in water by PAW when added in excess.

## 2. Materials and Methods

### 2.1. Isolation and Cultivation of Bacterial Strains and Isolates

The microorganisms used in this study are reference strains and environmental isolates. The reference strains were purchased from the German Collection of Microorganisms and Cell Cultures (Leibniz Institute DSMZ, Braunschweig, Germany) and originated from the American Type Culture Collection (ATCC). 

To obtain optimum growth, bacteria were cultivated on Luria Bertani (LB)-, R2A- (Roth, Karlsruhe, Germany) and Columbia blood agar plates (Oxoid, Wesel, Germany) at 37 °C for 24 h until well-grown single colonies were recognizable.

Environmental microorganisms were isolated as part of surface water monitoring and through the regular sampling of pipe water installations. Coliform bacteria were identified by growth on chromogenic coliform agar (CCA; Xebios, Düsseldorf, Germany) with blue-violet (*Escherichia coli*) and red (coliform) screening of colored colonies. Multi-drug resistant *E. coli* and coliform microorganisms were pre-screened by growth on chromogenic ESBL (Extended-Spectrum-β-Lactamase) plates (chromID ESBL agars; bioMérieux, Nürtingen, Germany) and identified by their differential appearance with green-blue coliform and red *E. coli* color. Cultivation of the environmental isolates on plates was carried out at 37 °C in each case for 24 h to 48 h. Isolates were singularized and subcultivated under identical conditions until pure and single colonies were visible. The ESBL phenotype was proved by subcultivation on ESBL plates. Isolates were identified to species level using the MALDI biotyping technique (Bruker Daltonik, Bremen, Germany) according to the manufacturer’s protocol. 

### 2.2. Generation and Use of Plasma-Activated Water (PAW)

The production of plasma-activated water (PAW) based on tap water of the Institute of Hygiene, Münster, that is characterized by a slightly alkaline pH (about pH 8) and a total hardness of 10.5 °dH, by an electrical conductivity of approximately 511 µS/cm and by an oxidation–reduction potential (ORP) of approximately 441 mV was described recently [32]. Water batches of approximately 600 mL were plasma-activated using a PAW lab unit (VitalFluid, Eindhoven, The Netherlands) consisting of a power modulator and plasma reactor system for high-yield PAW production. The system generates up to 80 kV dual-resonant high voltages pulses with an oscillation frequency up to 2 MHz, a pulse repetition rate up to 20 kHz, and an average power of 800 W continuous as described in detail [25]. During the plasma activation process, a gas-phase plasma arc in ambient air is generated above the water surface [25] associated with a massive decrease in pH and an increase in the oxidation potential [32]. The energy application and the generation time corresponded with electrical conductivity and pH [25]. The more energy and the longer the production time, the higher the electrical conductivity and the lower the pH become.

In this study, tap water was plasma-activated at different electric power levels with 90 W and 100 W for 10 min and 30 min as specified in each case. For each batch, freshly generated PAW was always checked by pH to a maximum value of 3.5, which was determined using a pH meter (Xylem Analytics, Weilheim, Germany), and the solution was stored for a maximum of 24 h at room temperature. Prior to bacterial inactivation studies, PAW was tested for sufficient activity to inactivate the control strain *E. coli* in each case. 

### 2.3. Kinetics of Bacterial Inactivation by PAW

In the first part of the experimental series, three different bacteria species were cultivated in LB broth (Roth, Karlsruhe, Germany) at 37 °C overnight with shaking. *E. coli* was selected as representative of Gram-negative reference strains, *Serratia fonticola* as a Gram-negative environmental isolate, and *Staphylococcus aureus* was chosen out of the Gram-positives. PAW, activated at 90 W and at 100 W, respectively, each for 10 min, was added in the same volume as the initial volume of the cell suspension. The mixture was incubated at room temperature for different periods up to 120 min as indicated. To track the kinetics of inactivation over time, PAW was neutralized by the application of a nine-fold volume of neutralization solution (comprising 3 L-α-phosphatidylcholine (0.3%), Tween 80 (3%), sodium thiosulfate (0.5%), L-histidine (0.1%), and saponine (3%) (Roth, Karlsruhe, Germany)), according to DIN EN 1040 which corresponds to a dilution by a factor of 10. The impact of the acidic pH alone was analyzed in acidic saline solution (pH 3.5). Prior to the suspension of bacteria in saline (0.9% (*w*/*v*) NaCl; Roth, Germany), cells were washed and harvested via centrifugation (2.000× *g*; 10 min). The cell density of the initial solution was adjusted to 1−5 × 10^8^ cells mL^−1^ proved by spotting ten-fold dilution series in 10 µL volumes in triplicate on LB agar plates. After PAW treatment, bacterial survival was analyzed by spotting aliquots of 10 µL volumes in ten-fold dilution series in triplicate on agar plates followed by incubation at 37 °C for 24 h. Visible single colony-forming units (CFUs) were counted out of each evaluable dilution and the cell count/mL was determined. It should be noted that the detection limit for this method is around 2 log_10_ CFU/mL but is sufficient for all these tests.

### 2.4. Effect of PAW on Various Bacterial Species

The nature and composition of the solution, in which the bacteria are suspended prior to PAW treatment, and the cell density may have an impact on slight or delayed reduction in living cells and inactivation. Therefore, the impact of the matrix solution, in which the microorganisms are present, was analyzed. To minimize disruptive and stressful conditions such as, e.g., centrifugation steps, single colonies, well-grown on agar plates overnight, were initially suspended in saline. The density was set between 10^5^ and 10^7^ CFUs mL^−1^ which was verified by spotting in the dilution series at 10 µL volumes in triplicates on LB agar plates followed by incubation at 37 °C for 24 h. CFUs were counted and the cell number per mL was determined. The initial cell suspensions were diluted tenfold either in sterilized tap water or in sterile saline solution as indicated. In these assays, PAW was generally produced at 90 W for 30 min. These settings were chosen because the activation at 90 W was sufficient for the inactivation of high cell numbers if an adequate activation time of 30 min was used while the activation time of only 10 min showed limitations. Additionally, the survival rates could be determined more reliably than with PAW activated at 100 W.

PAW was added to the microbiological dilutions in the same or twice the volume of the initial cell suspension followed by incubation periods as indicated. In this experimental approach, the reaction treatments were not neutralized in order to avoid a high dilution effect on the one hand and to be able to recognize long-term effects on the other hand.

Serial dilutions were spotted in 10 µL volumes in triplicate on agar plates followed by incubation at 37 °C for 24 h. Bacterial survival was assessed by CFU counting and calculating the cell density in CFU/mL.

### 2.5. Data Analysis

As the assays were always carried out at the PAW’s effect limit, the test preparations were repeated at least two to three times for each individual bacterium. In some test series, the limitation effect was not clearly recognizable, i.e., when PAW had an inactivating effect. Therefore, the results shown here represent one exemplary test approach with the worst outcome using the mean value from the triplicate plating determination. The total number was determined from the countable dilutions as arithmetic means (±standard deviation).

## 3. Results

The effect of PAW on microorganisms in aqueous solutions, associated with a dilution effect, was assessed on two different aspects: firstly, the bacterial survival rates were analyzed after contact with PAW, activated at different levels of power, and with various periods. Secondly, the influence of the dilution solution, in which the bacteria were suspended, was analyzed on the effect of survival after exposure to PAW.

### 3.1. Impact of Plasma Activation Settings on Cell Reduction in Gram-Negative and Gram-Positive Microorganisms over Time

**Figure 1:**
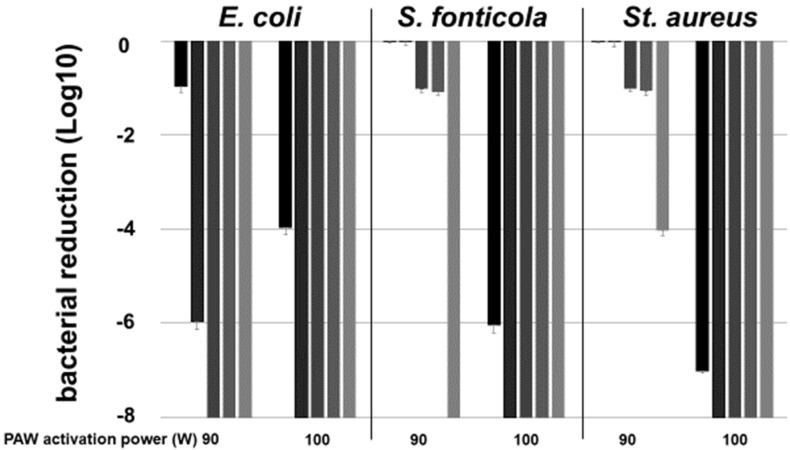
Impact of PAW activation on CFU reduction. PAW, activated at 90 and 100 Watt each for 10 min as indicated, was added at identical volumes to suspensions of *E. coli*, *Serratia* (*S.*) *fonticola*, and *Staphylococcus* (*St.*) *aureus* each at initial concentrations of 8 Log10 mL^−1^. The bacterial reduction is given in Log10-stages after 5 min (dark black columns), 10 min (black columns), 30 min (dark grey columns), 60 min (grey columns), and 120 min (light grey columns). The plate counting and the calculation of CFU mL^−1^ was carried out in triplicate, and the standard deviation is given for the reduction levels.

To determine the PAW activation conditions’ impact on the bacteriological inactivation rates over time, combinations of PAW generation power and microbial contact periods were analyzed. Microorganisms at the high concentration of 8 Log10 cells mL^−1^ in saline, indicating a massive contamination, were exposed to PAW, either activated at 90 W or at 100 W each for 10 min, for various time periods up to 120 min (Figure 1).

After 10 min incubation at the latest, all microorganisms were no longer cultivable at the high PAW activation level of 100 W/10 min. The lower PAW activation (90 W/10 min) resulted in limitations in bacterial inactivation. No survival of the Gram-negative microorganisms *E. coli* and *S. fonticola* was seen after 30 and 120 min, respectively, while *S. aureus* only decreased by 4 Log10-levels after 120 min of contact time. In summary, PAW inactivated the fecal contaminant *E. coli*, the environmental isolate *S. fonticola,* and the Gram-positive bacterium *S. aureus* in a PAW activation power and a time-dependent manner (Figure 1).

### 3.2. Impact of the Aquatic Matrix on Microbiological Reduction and Inactivation by PAW

The efficiency of bacterial inactivation by PAW was examined based on various bacterial species and different cell densities as well as on the resuspension and the dilution media, in which the microorganisms were present during incubation with PAW. In order to enable a clear differentiation of the limits between still-surviving and already-dead in the different bacterial species, the activation of PAW was set to 90 W for 30 min. We found that the dilution solution, in which the bacteria are present, was of paramount importance for the subsequent inactivation of microorganisms by PAW. Bacteria at concentrations of 10^5^ to 10^7^ mL^−1^, as indicated in Table 1, suspended in saline were exposed to PAW in a ratio of 1:1. The data in Table 1 always represent the highest cell densities used in the assays and the corresponding cell reductions. The Gram-negative reference strains and environmental isolates were completely inactivated after 30 min of contact time evidenced by failing to grow on plates (Table 1). 

As expected, there was no difference between antibiotic-resistant and antibiotic-susceptible isolates as well. Additionally, no colonies of the environmental isolates *Sphingomonas paucimobilis* and *Stenotrophomonas maltophilia* (Table 1 and as described previously [39]) came up, as well as the further analyzed bacteria of *Brevundimonas diminuta*, *Burkholderia* spp. and *Buttiauxella* spp. failing to grow under these conditions.

In contrast, the Gram-positive bacteria proved to be more robust to this treatment (Table 1). The enterococcal strains of the investigated species *E. faecalis* and *E. faecium* showed a high survival rate in saline, and the total bacterial counts decreased only slightly by 0.4 and 0.5 Log10-levels at 10^6^ mL^−1^ cells, respectively, after PAW exposure. The strains of *S. epidermidis* and *S. aureus* were more susceptible. A complete loss of cultivation was observable for *S. aureus* at an initial cell density of 10^5^ cells mL^−1^, while very few *S. epidermidis* cells survived at a higher density of 10^7^ cells mL^−1^ but reduced in survival by 4.9 Log10 stages (Table 1). The inactivation efficiency was lower for Gram-positive cells compared with Gram-negative cells at high cell densities, although the successful inactivation effect of PAW was evident using lower cell numbers.

**Figure 2:**
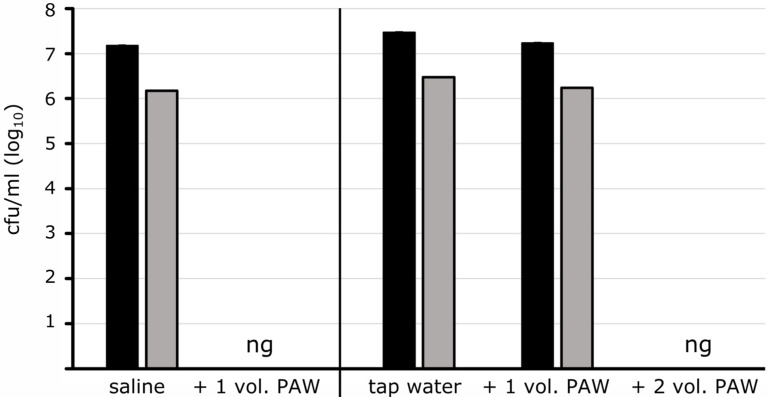
Susceptibility of *Serratia fonticola* in saline and tap water to PAW. *S. fonticola* was suspended in saline (left) and in tap water (right) with counts of 10^7^ (black columns) and 10^6^ (grey columns) cells/mL. PAW was added in equal (+1 vol. PAW) and double volume ratios (+2 vol. PAW) followed by a contact time of 30 min. Aliquots were transferred to plates to check survival. No growth (ng) was seen when saline-suspended cells were incubated with the equal volume of PAW. In contrast, cells in tap water showed only a minimal reduction under these conditions, while no CFUs came up with the double PAW volume. Plate counting, given as CFU mL^−1^, was carried out in triplicate (±standard deviation).

In the following, the PAW effect on bacteria was analyzed when the microorganisms were present in tap water. This represents aqueous environments including waterborne bacteria and contaminants that exist and live in water. The inactivation of Gram-negative reference strains and the environmental isolates at cell densities of 10^5^ to 10^7^ mL^−1^ was low when PAW (90 W, 30 min) was added in the same unit of volume to bacteria in tap water (Table 1). A complete inactivation after 30 min of contact time was only achieved with higher dilution levels and lower cell concentrations. However, when PAW (90 W, 30 min) was present in excess as with double the volume, the disinfection effect was observable (Figure 2) and was verified for all analyzed Gram-negative microorganisms (Table 1). Only *Citrobacter freundii* showed some survival as its cell concentration reduced by 2 Log10-levels at the highest initial concentration of 10^5^ mL^−1^, but was completely inactivated at lower cell concentrations.

In water, the Gram-positives were highly robust to this treatment, even with double the amount of PAW added. The inactivation of enterococci was very low after 30 min (Table 1) proving them as highly robust environmental bacteria. *S. aureus* numbers with the highest cell density of 10^5^ mL^−1^ decreased by 2.4 Log10-levels with one and by 3.5 Log10-levels with doubled PAW volumes, respectively. Starting with 10^4^ cells mL^−1^ growth failed totally with one and double PAW volumes. *S. epidermidis* reduced by 2.6 and 2.7 Log10-levels at densities of 10^7^ cells mL^−1^ (Table 1) and at densities of 10^6^ cells mL^−1^, respectively, with PAW at the identical volume while no colonies came up with the doubled PAW volume added (Table 1).

**Figure 3:**
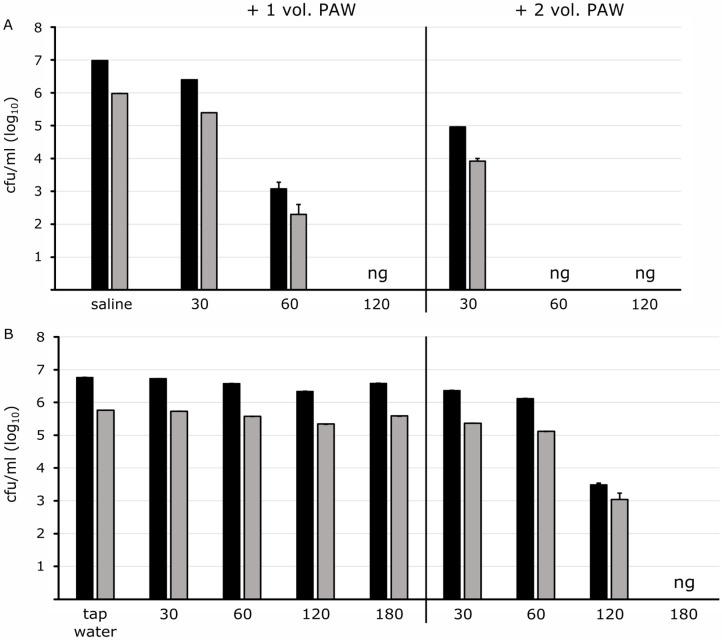
Survival kinetics of *E. faecalis*. *E. faecalis* was suspended in saline (**A**) and tap water (**B**). Cells were confronted with PAW (activated 90 W/30 min) in equal (+1 vol. PAW) and double volumes (+2 vol. PAW) for time periods as indicated prior to spotting aliquots of dilutions on agar plates. Regardless of the cell densities of 10^7^ (black columns) and 10^6^ cells/mL (grey columns), the cell number only reduced minimal with an equal volume ratio in tap water over time while it reduced and came down to no growth (ng) after 60 and 120 min, respectively, when suspended in saline. PAW added in surplus (+2 vol. PAW) inactivated the bacteria more efficiently indicated by no growth on plates (ng). Data represent the mean values from one set-up in the triple approach with standard deviation in appropriate dilutions.

To find out if Gram-positive bacteria are generally robust or tolerant to PAW, the contact time to PAW was extended. The fecal pathogen *E. faecalis* suspended in saline showed a reduction of 0.4 Log10-levels after 30 min and 3.5 Log10-levels after 60 min of contact time with PAW added in the same volume (Figure 3A). Complete inactivation was achieved after 120 min.

Doubling the PAW volume improved the bacterial inactivation efficiency. A decrease of nearly 2 Log10-levels was achieved after 30 min, and after 60 min no growth was detectable anymore (Figure 3A). *E. faecium* showed robustness as well: in saline, a reduction of 0.5 and 2.6 Log10-levels could be achieved after 30 min and 60 min, respectively, with the same volume of PAW added. Twice the volume of PAW led to a reduction of 1.1 and 3.3 Log10-levels after 30 min and 60 min, respectively. No bacterial growth was detected after 120 min under these conditions.

When diluted in water, the enterococcal strains survived in a one-to-one volume with PAW for more than 180 min (Figure 3B) and even after a 24 h incubation period. Only the double PAW volume unit added to the bacterial suspension caused a decrease in the cultivable cells starting after a prolonged exposure time of 120 min. No living microorganisms were noticed after 180 min (Figure 3B).

The staphylococcal strains included quickly reduced when diluted in saline and were completely inactivated after 30 min and 60 min, respectively. High cell densities of *S. aureus* in tap water reduced continuously over time, and no growth was visible after 120 min incubation with a one-to-one PAW volume added while *S. epidermidis* proved to be somewhat more robust with complete inactivation after 180 min of exposure time. When twice the PAW volume was added, inactivation was accelerated and observed after an incubation time of 30 min for *S. epidermidis* and 60 min for *S. aureus*.

## 4. Discussion

While maintaining effectiveness, there is a great need for new easy-to-use disinfection technologies that do not generate undesirable byproducts. PAW has proved to be a novel technology for the inactivation of microorganisms, and several studies have demonstrated the successful application of PAW; however, this was always achieved by adding PAW in high excess or directly by immersing food in PAW [6,18,36]. The generation of PAW is accompanied by an increase in RONS associated with a significant decrease in the pH [6,18,26,40,41]. For bacterial inactivation, a maximal boundary at pH 4.7 was reported [42].

Plasma-activated tap water, activated at 90 W for 30 min, resulted in a pH below 3.5. The acidic pH is considered indispensable for the inactivation process and essential for biocidal action [6,18,42] but is mainly the result of the formation of RONS, which are responsible for the antibacterial effect. When storing PAW, the pH value hardly changes, but the effect of the RONS diminishes, in some cases in conjunction with diminishing antimicrobial properties [43]. In our test series, the antimicrobial effect was still present after up to 24 h of storage. The conductivity, indicating the presence of ions, as well as the oxidizability in plasma-activated tap water rose markedly indicating physical and chemical changes in the water associated with an increase in oxidative effective substances [32]. The conductivity of the generated PAW originating from the institute’s tap water increased from 511 µS/cm to 1045 µS/cm but reduced in a one-to-one and a one-to-two dilution to 600 and 687 µS/cm, respectively [32]. The decrease in pH after plasma activation interdepends inversely on the electrical conductivity increase [44]. When tap water was added to PAW, the electrical conductivity decreased in correlation with an increase in pH. The ORP, revealing the ability to oxidize a solution, increased and seems to have an important impact on the bactericidal effect of PAW. Thereby, hydrogen peroxide is considered the main substance responsible for the ORP value [44]. In our hands, the ORP in PAW–water mixes increased from 441 mV to 753 and 745 mV with one- and two-volume PAW units, respectively, and remained at this level (724 mV) in saline [32].

### 4.1. Antimicrobial Efficiency of Plasma-Activated Water

Our study aimed to analyze the impact of PAW on the inactivation of microorganisms living in aqueous environments because the volume of PAW added to aqueous suspensions is limited. Hence, we investigated the effect of low and limited PAW volume addition in order to consider the dilution effects while maintaining simultaneous sufficient microbiological inactivation. Additionally, possible material-damaging effects due to oxidative active substances can be minimized by using lower PAW volumes or by using PAW with less oxidizing and damaging substances.

The bactericidal effect correlated with the increase in the electrical power needed to generate PAW and the plasma-activation duration. The activation of tap water at 100 W was more efficient than at 90 W, regardless of the respective 10 min activation time, probably due to more activation energy. The more intensively plasma-activated, the better the antimicrobial effect. This result indicated that more oxygen and nitrogen reactive species are formed at higher voltages and longer activation times. Several studies demonstrated increased ORP values with prolonged plasma activation times and treatments [22,36,45,46].

The activation efficiency of PAW depends both on the activation process and on the device itself, but also on the processing parameters. The disinfection potential of PAW depends on the applied voltage, the plasma treatment time for PAW generation [47], and the treatment models on indirect or direct ways [48] of feeding gases and air flow [49,50]. In addition, the water quality and chemistry may have an impact on the generation of RONS in PAW, whose quantities can increase or minimize the antimicrobial efficacy. Little is known about the chemical and physical quality and subsequent activation level of different tap water sources used for plasma–water activation because most studies have been carried out with defined water qualities in a laboratory scale. However, several abiotic parameters may decrease the PAW activity such as the presence of organic materials [21,46] as well as the storage temperature [30]. In this study, we used tap water from our institute resulting in a jump down from a neutral/slightly alkaline pH to an acidic solution after activation using our settings. However, it was reported that this pH decrease failed from another source of tap water [51], and water hardness affected the active components of PAW [52]. Sanitation, cleaning, and washing processes need sufficient volumes of plasma-activated water. Therefore, it is proposed to adapt the technology by electric power and activation time to existing water sources to achieve the best inactivation quality as well as to optimize the contact time to microorganisms accordingly. When the water sources and quality change, a repeated control and optimization of the settings are recommended.

### 4.2. Impact of the Microbial Nature to Inactivation by PAW

The mechanisms of PAW on microorganisms are oxidative stress and a physical effect. Reactive oxidative substances have a destructive effect on the cell membrane, with some substances breaking structurally relevant bridge bonds of the peptidoglycan layer [53], depolarizing the cell membrane, and oxidizing lipids and proteins [33,34]. The extent of cell damage depends not only on the activation power of PAW, but also on the microbiological characteristics such as vegetative cells or spores and the presence as planktonic cells or in the biofilm [6]. These investigations were based on PAW generation at 90 W and 30 min activation for successful but also borderline bacterial inactivation after 30 min of contact time. Microbiological analyses resulted in the rapid inactivation of selected Gram-negative and delayed but sufficient inactivation of Gram-positive microorganisms with PAW in limited volume addition. The double PAW volume unit to one volume unit of bacterial suspension in tap water was sufficient for total inactivation. However, the contact times needed for complete inactivation varied between Gram-negative and Gram-positive microorganisms. The Gram-negatives, all belonging to mammalian-adapted (fecal) and environmental *Enterobacterales*, as well as the waterborne microorganisms, failed in growth after 30 min of incubation. The Gram-positives demonstrated a higher robustness and the inactivation rate was limited under these conditions. However, prolonged contact time to PAW resulted in an increasing reduction in *Enterococci* and *Staphylococci* and inactivation could be observed after an incubation time of 180 min at the latest. In general, Gram-positive microorganisms clearly showed a higher stability than the Gram-negative ones. These results, consistent with previous findings [6,17,18,19,54], are explainable by means of the different cell wall nature. The thicker cell wall in Gram-positive bacteria appears to provide protection against PAW, as it possesses multiple layers on the envelope compared with the Gram-negatives. This results in a higher stability of the cell structure and protection from the diffusion of oxidative effective substances [6,18]. However, the membrane composition cannot be the sole characteristic of bacterial susceptibility or robustness to PAW. The Gram-positive species *Enterococcus faecalis*, indicator for detection of fecal inputs in water lines, demonstrated a higher stability to PAW treatment than the Gram-positive human skin bacteria of the *Staphylococcus* genus. Additionally, it should be considered that low cell densities were inactivated more easily than high densities with low inactivation rates. The ratio of reactive substances to a single bacterium is significantly reduced at high cell densities, and several microorganisms tend to form aggregates and layers [16,55].

Interestingly, the environmental matrix solution, in which the bacteria were present during PAW treatment, had a decisive impact on the successful inactivation efficacy. The effect of PAW was usually studied against bacteria that had been suspended in saline [40] and phosphate-buffered saline [6,18]. In saline, the Gram-negative microorganisms showed a high susceptibility to PAW treatment. The application of one volume unit was sufficient for complete inactivation. When bacteria were present in tap water, however, twice the volume had to be added for inactivation. This phenomenon of lower stability in saline compared with enhanced stability in tap water has also been observed with Gram-positive bacteria. We suppose that a bacterium in each environmental medium immediately responds with appropriate physiological adaptations with the consequence of increasing or decreasing stability, regardless of cell wall composition. This assumption of the current physiological state is supported by the observation of microorganisms in different growth states. After PAW treatment, bacteria in the exponential phase were considerably reduced to a greater extent compared with cells in the stationary phase [56]. However, it should also be considered that by adding PAW to saline with high salt concentrations, further chemical processes may take place and thus more and different RONS may be formed. Thus, the antimicrobial effect appears more efficient. 

## 5. Conclusions

In conclusion, the composition of PAW and its disinfecting properties depend strongly on the way in which the water is activated. The electrical power, the type of plasma (thermal, non-thermal), treatment time of the water, water composition, and the water–gas interface play an important role in this process. It is always possible to treat water in such a way that a more powerful disinfecting effect is obtained by varying the mentioned parameters. In this study, two fixed PAW process settings were used that produced the results described; other results will be achieved at other plasma settings.

The activation by plasma as a novel technology and PAW generated from tap water as an emerging biocidal agent have gained attraction and can be used in sanitation, washing, and cleaning processes with wide applications. The results of the study demonstrate that PAW is an effective antimicrobial agent even when used in small volumes or as dilutions. This has the advantage that, on the one hand, a smaller volume can be used in washing and cleaning processes for efficient microbiological inactivation, and, on the other hand, the material-damaging effect can be minimized by diluting the oxidative active substances at a very low pH.

Gram-negative bacteria were highly susceptible to PAW treatment in low volumes, while the counts of Gram-positive microorganisms reduced with longer contact times, with extended plasma activation, and with higher PAW volumes. Microorganisms present in saline attained a high susceptibility, while existence in tap water indicated robustness and increased survival. However, tap water is the target in which the microorganisms to be eradicated exist and live, and the application of the technology is also related to the use of water processes. These different antimicrobial efficiencies can be due to the possible physiological change in the bacteria from susceptible in saline to a robust condition in tap water. On the other hand, the PAW conditions may have an influence as chemical and physical parameters change depending on addition to saline or tap water.

## Figures and Tables

**Table 1:** Impact of plasma-activated water (PAW) on bacterial strains and isolates suspended in different solutions.

Gram-Negative	1 vol. Saline + 1 vol. PAW *			1 vol. Tap Water + 1 vol. PAW *			1 vol. Tap Water + 2 vol. PAW *		
Strain/Isolate	Pre-Incubation	Post-Incubation	Reduction (Log10)	Pre- Incubation	Post- Incubation	Reduction (Log10)	Pre-Incubation	Post-Incubation	Reduction (Log10)
*Citrobacter freundii* ATCC43864	7.7 × 10^5^ (±3.1 ×10^4^)	ngr	5.9	1.3 × 10^5^ (±6.0 × 10^4^)	1.0 × 10^3^ (±3.5 × 10^2^)	2.1	1.0 × 10^5^ (±2.6 × 10^4^)	1.0 × 10^3^ (±3.5 × 10^2^)	2.0
*Enterobacter cloacae* ATCC13047	-	-	-	5.9 × 10^5^ (±5.3 × 10^4^)	6.0 × 10^5^ (±6.1 × 10^4^)	0	1.9 × 10^5^ (±3.0 × 10^4^)	ngr	5.3
*E. coli* ATCC25922	1.8 × 10^7^ (±5.0 × 10^6^)	ngr	7.3	2.8 × 10^7^ (±7.0 × 10^6^)	1.7 × 10^7^ (±3.1 × 10^6^)	0.2	2.9 × 10^7^ (±4.6 × 10^6^)	ngr	7.5
*E. coli* A2904-1-1-12/94 **/***	1 × 10^7^ (±0)	ngr	7.0	4.4 × 10^7^ (±6.1 × 10^6^)	2.2 × 10^7^ (±4.2 × 10^6^)	0.3	3.4 × 10^7^ (±9.6 × 10^6^)	ngr	7.5
*Klebsiella pneumoniae* ATCC13883	1 × 10^7^ (±0)	ngr	7.0	4.3 × 10^5^ (±6.0 × 10^4^)	2.5 × 10^6^ (±2.0 × 10^5^)	0	2.5 × 10^5^ (±9.2 × 10^4^)	ngr	5.4
*Lelliottia amnigena* 670 **	2.2 × 10^7^ (±1.2 × 10^7^)	ngr	7.3	1.9 × 10^7^ (±2.1 × 10^6^)	1.0 × 10^6^ (±0.8 × 10^5^)	1.3	1.9 × 10^7^ (±1.2 × 10^6^)	ngr	7.3
*Lelliottia amnigena* 671 **	2.1 × 10^7^ (±2.3 × 10^6^)	ngr	7.3	1.5 × 10^7^ (±4.7 × 10^5^)	1.0 × 10^7^ (±0.8 × 10^6^)	0.2	1.5 × 10^7^ (±4.7 × 10^5^)	ngr	7.2
*Pseudomonas aeruginosa* ATCC27853	3.2 × 10^7^ (±7.4 × 10^6^)	ngr	7.5	2.4 × 10^4^ (±5.3 × 10^3^)	5.7 × 10^4^ (±6.1 × 10^3^)	0	3.0 × 10^4^ (±1.2 × 10^4^)	ngr	4.5
*Serratia fonticola* DSM14576	1 × 10^7^ (±0)	ngr	7.0	-	-	-	-	-	-
*Serratia fonticola* 612 **	1.5 × 10^7^ (±4.2 × 10^6^)	ngr	7.2	1.6 × 10^7^ (±8.3 × 10^6^)	5.2 × 10^6^ (±1.1 × 10^6^)	0.5	2.2 × 10^7^ (±1.7 × 10^6^)	ngr	7.3
*Serratia fonticola* 624 **	1.5 × 10^7^ (±1.1 × 10^7^)	ngr	7.2	1.9 × 10^7^ (±1.2 × 10^6^)	1.7 × 10^7^ (±4.2 × 10^6^)	0	1.8 × 10^7^ (±3.0 × 10^6^)	ngr	7.3
*Serratia fonticola* 9–65 **/***	-	-	-	1.8 × 10^7^ (±7.2 × 10^6^)	2.5 × 10^7^ (±4.2 × 10^6^)	0	1.2 × 10^7^ (±1.8 × 10^6^)	ngr	7.1
*Sphingomonas paucimobilis* 549 **/***	-	-	-	7.0 × 10^6^ (±1.1 × 10^6^)	6.6 × 10^6^ (±5.3 × 10^5^)	0	7.0 × 10^6^ (±1.1 × 10^6^)	ngr	7.1
*Stenotrophomonas maltophilia* 650 **/***	3.5 × 10^7^ (±8.7 × 10^6^)	ngr	7.5	3.6 × 10^7^ (±3.5 × 10^6^)	1.7 × 10^7^ (±8.3 × 10^6^)	0.3	3.6 × 10^7^ (±3.5 × 10^6^)	ngr	7.6
Gram-positive									
*Enterococcus faecalis* ATCC19434	6.2 × 10^6^ (±7.2 × 10^5^)	2.5 × 10^6^ (±3.3 × 10^5^)	0.4	8.4 × 10^6^ (±5.0 × 10^5^)	5.4 × 10^6^ (±8.7 × 10^5^)	0.2	5.5 × 10^6^ (±1.7 × 10^5^)	2.3 × 10^6^ (±2.3 × 10^5^)	0.4
*Enterococcus faecium* HYMS015	3.3 × 10^6^ (±7.5 × 10^5^)	9.5 × 10^5^ (±1.0 × 10^5^)	0.5	3.2 × 10^6^ (±9.0 × 10^5^)	3.2 × 10^6^ (±7.6 × 10^5^)	0	3.1 × 10^6^ (±8.7 × 10^5^)	1.8 × 10^6^ (±1.1 × 10^5^)	0.2
*Staphylococcus aureus* ATCC6538	5.6 × 10^5^ (±1.3 × 10^5^)	ngr	5.8	4.1 × 10^5^ (±1.6 × 10^5^)	1.6 × 10^3^ (±2.0 × 10^2^)	2.4	3.2 × 10^5^ (±1.7 × 10^4^)	1.0 × 10^2^ (±1.7 × 10^2^)	3.5
*Staphylococcus epidermidis* ATCC12228	1.1 × 10^7^ (±1.5 × 10^6^)	1.3 × 10^2^ (±1.2 × 10^2^)	4.9	1.1 × 10^7^ (±1.6 × 10^6^)	2.4 × 10^4^ (±8.8 × 10^3^)	2.6	8.6 × 10^6^ (±1.4 × 10^6^)	ngr	6.9

* PAW (90 W/30 min) was added in 1 or 2 volume units to bacterial saline and tap water. The reduction in cell counts (in Log10-units) and complete inactivation (no growth; ngr) are indicated after 30 min of contact time. Numbers in brackets represent the standard deviations. Few experimental set-ups were not carried out (-). ** environmental isolates. *** Isolates that are resistant to one or more β-lactam antibiotics.

## Data Availability

The data presented in this study are available on request from the corresponding author.
